# Gap junction protein beta 5 interacts with Gαi3 to promote Akt activation and cervical cancer cell growth

**DOI:** 10.1038/s41419-025-07768-w

**Published:** 2025-06-19

**Authors:** Ping Li, Jie Chen, Juan Wang, Tianbo Liu

**Affiliations:** 1 Department of Radiotherapy and Oncology, Affiliated Kunshan Hospital of Jiangsu University, Kunshan, China; 2 Department of Gynecology, Harbin Medical University Cancer Hospital, Harbin, China; 3 Department of Obstetrics and Gynecology, The First Affiliated Hospital of Soochow University, Suzhou, China

**Keywords:** Cervical cancer, Cervical cancer

## Abstract

Identifying novel therapeutic targets for cervical cancer is crucial for improving patient outcomes and reducing the global burden of this disease. Gap junction protein beta 5 (GJB5) is a member of the connexin family of proteins involved in cell-to-cell communication. This study investigated GJB5’s expression and functional significance in cervical cancer. Analysis of The Cancer Genome Atlas (TCGA) data demonstrated significantly increased *GJB5* mRNA expression in cervical cancer tissues compared to normal cervical epithelium. Moreover, high *GJB5* expression correlated with reduced overall survival and other adverse clinical outcomes. Single-cell RNA sequencing corroborated *GJB5* overexpression within the malignant tumor cell population. The downregulation of GJB5 through shRNA or CRISPR/Cas9 gene knockout techniques significantly impaired the viability, proliferation, and migratory capacity of cervical cancer cells, while concurrently inducing apoptotic processes. Conversely, the forced overexpression of GJB5 resulted in enhanced malignant behaviors. Investigations into the underlying mechanisms revealed that GJB5 is integral to the activation of the Akt-mTOR (mammalian target of rapamycin) signaling pathway. GJB5 knockdown or knockout led to diminished phosphorylation of Akt and S6 kinase, whereas GJB5 overexpression correlated with increased Akt-mTOR signaling in primary human cervical cancer cells. Additionally, we identified a novel interaction between GJB5 and the Gαi3 (G alpha inhibitory protein 3), underscoring the crucial role of GJB5 in mediating Akt activation via Gαi3. In vivo studies utilizing xenograft models provided further evidence for the oncogenic function of GJB5. The knockdown of GJB5 resulted in a marked reduction in the growth of cervical cancer xenografts. Observations of proliferation arrest, inactivation of the Akt-mTOR pathway, and the induction of apoptosis were noted in GJB5-depleted cervical cancer xenograft tissues. Collectively, these findings underscore GJB5 as a key oncogenic driver in cervical cancer and indicate that targeting GJB5 could be a promising therapeutic approach for this disease.

## Introduction

Cervical cancer, a significant global health burden, primarily arises from persistent infection with high-risk human papillomavirus (HPV) genotypes [1, 2]. This malignancy disproportionately affects women in low- and middle-income countries due to limited access to screening and vaccination programs [3, 4]. There are an estimated 660,000 new cases and 350,000 deaths from cervical cancer globally, underscoring the need for effective prevention strategies [5, 6]. Cervical cancer predominantly presents as squamous cell carcinoma (SCC) or adenocarcinoma, with the former accounting for about 90% of cases, characterized by distinct cellular atypia and invasive growth patterns [1]. Current treatment paradigms encompass surgical intervention, radiotherapy, and chemotherapy, with treatment strategies tailored to the stage of disease progression [7]. However, challenges such as late-stage diagnosis, disparities in healthcare access, and suboptimal HPV vaccine uptake hinder effective management [8]. Consequently, continued research aimed at improving early detection, refining therapeutic approaches, and enhancing outcomes for affected populations remains crucial [1, 2].

Current targeted therapies for cervical cancer primarily encompass monoclonal antibodies [9–12], such as bevacizumab [13], which inhibits vascular endothelial growth factor (VEGF), and immune checkpoint inhibitors like pembrolizumab and nivolumab [14], which enhance anti-tumor T-cell immunity by blocking programmed cell death protein 1 (PD-1) [15, 16]. While these agents have demonstrated promising clinical efficacy in patients with recurrent or metastatic disease, resulting in improved progression-free and overall survival rates in select cancer populations, several limitations persist [10, 11, 17]. These include inter-patient variability in treatment response, the potential for immune-related adverse events, and the emergence of treatment resistance over time [10, 11, 17]. Furthermore, the current reliance on a limited number of therapeutic targets underscores the critical need to identify and validate novel molecular and genetic targets to expand the therapeutic armamentarium for cervical cancer [9–12]. This pursuit is essential for developing more personalized and effective treatment strategies that can improve outcomes for all patients. It is also the research focus of our group [18].

Gap junction protein beta-5 (GJB5), also known as connexin 31.1, is a member of the connexin family of proteins that form gap junctions, intercellular channels facilitating direct communication between adjacent cells [19, 20]. It is primarily expressed in the inner ear, skin, and various epithelial tissues, playing an important role in maintaining cellular homeostasis and tissue integrity [21–24]. GJB5 contributes to the formation of gap junction channels that allow the passage of ions and small molecules between adjacent cells, which is essential for processes such as cellular signaling and metabolic cooperation [21–25]. The mechanism of GJB5 involves the assembly of connexin proteins into hexameric structures, or connexons, that dock with those from neighboring cells to form functional gap junctions. GJB5 is defective in forming functional gap junctions on its own, but its interaction with co-expressed connexins like Cx43 can enhance its assembly into gap junctions and improve intercellular signaling [25]. Studies have explored other functions of GJB5. Deficiency of GJB5 impaired the differentiation of trophoblast stem cells and hindered placental development [21, 26]. GJB5 deficiency in knockout mice increased exploratory behavior and impaired object recognition, indicating its role in learning and memory [27].

The intercellular communication processes by GJB5 are important for epidermal differentiation and the sensing of environmental stimuli [21–24]. GJB5’s link to epidermal differentiation is particularly relevant as cervical cancer frequently originates in the squamous epithelium, which shares characteristics with epidermal tissue [1]. Furthermore, pan-cancer analyses indicate variable *GJB5* expression across malignancies, with notable reports of elevated *GJB5* mRNA expression in cervical squamous cell carcinoma [28]. This distinct expression profile in cervical squamous cell carcinoma, alongside its fundamental role in modulating gap junction and intercellular communication [21–24], suggest a potential involvement in cervical carcinogenesis. This study aims to investigate the expression, functional role, and potential underlying mechanisms of GJB5 in cervical cancer, as these aspects remain largely unexplored.

## Materials and methods

### Reagents and antibodies

Reagents and antibodies were obtained from the following sources: Puromycin, cell culture media, serum FBS, Matrigel matrix, polybrene, and CCK-8 were purchased from Sigma-Aldrich (St. Louis, MO). Terminal deoxynucleotidyl transferase dUTP nick end labeling (TUNEL), 5-ethynyl-2’-deoxyuridine (EdU), 4’,6-diamidino-2-phenylindole (DAPI), and JC-1 were acquired from Thermo Fisher Scientific (Invitrogen Thermo Fisher Scientific, Carlsbad, CA). All primary antibodies utilized in this study were sourced from Cell Signaling Technology (Danvers, MA, USA) and Thermo Fisher Scientific (Invitrogen, Suzhou, China). The nucleotide sequences and PCR primers were synthesized by Genechem (Shanghai, China).

### Cells

As previously detailed [18, 29], cervical cancer tissues and neighboring epithelial tissues were fragmented and digested with collagenase type I and dispase II (Sigma). The cells were washed, centrifuged, and incubated in a complete medium supplemented with penicillin/streptomycin and DNase (500 U). Following filtration and re-suspension, primary cervical cancer cells (“priCC-1”, “priCC-2”, and “priCC-3”, from three individuals) and cervical epithelial cells (“priCEpi-1” and “priCEpi-2”, from two individuals) were cultivated in the described medium [29]. HeLa229 and Caski cervical cancer cell lines were obtained from the Cell Bank of the Institute of Biological Science at CAS (Shanghai, China). All procedures involving human cells were authorized by the Ethics Committee of The Affiliated Kunshan Hospital of Jiangsu University and conformed to the principles of the Helsinki Declaration.

### Human tissues

Cervical cancer tissues and corresponding paracancerous normal epithelial tissues were obtained from a cohort of twenty patients (*n* = 20) at the Affiliated Hospitals of Soochow University following the acquisition of written-informed consent from each participant. Pertinent patient-specific details have been previously reported [29]. All of the cancers are identified as squamous cell carcinomas [29]. Fresh tissue lysates were subjected to Western blotting analysis and quantitative real-time PCR (qPCR) for further investigation. All experimental procedures were granted approval by the Ethics Committee of the Affiliated Kunshan Hospital of Jiangsu University and were conducted in strict adherence to the ethical guidelines outlined in the Helsinki Declaration.

### Immunohistochemistry (IHC)

Immunohistochemistry (IHC) was conducted on paraffin-embedded tissue sections with a thickness of 4 μm. After standard de-paraffinization and rehydration, antigen retrieval was performed using citrate buffer. To inhibit endogenous peroxidase activity, the sections were treated with hydrogen peroxide. The primary antibody was then applied and incubated for 10 h. Following this, a secondary antibody and streptavidin-horseradish peroxidase conjugate were introduced, each incubated for 2 h. Finally, diaminobenzidine (DAB) was utilized to visualize the expression of the target antigen.

### Western blotting and co-immunoprecipitation (Co-IP) assays

In brief, protein extracts (30–40 µg) from cellular and tissue lysates were resolved using SDS-PAGE gels (10–15%) and transferred onto PVDF membranes. Following a blocking step, membranes were incubated with primary antibodies overnight at 4 °C, followed by incubation with designated secondary antibodies for 45 min at ambient temperature. Protein bands were visualized using enhanced chemiluminescence (ECL) and quantified using ImageJ software. The specific protocols for Co-IP have been detailed elsewhere [30]. Uncropped blot images are presented in Fig. S1.

### qPCR

In brief, RNA was isolated from cells and tissues using TRIzol (Biyuntian, Wuxi, China), reverse transcribed into complementary DNA (cDNA) with a Takara PCR amplification kit (Beijing, China), and quantified using qPCR with SYBR Green PCR Master Mixes (Beijing, China) on the ABI-7900 system. *GAPDH* was employed as the reference gene, and relative gene expression was determined using the 2^−ΔΔCt^ method. Primers were obtained from Genechem.

### *GJB5* gene silencing

Two different short hairpin RNAs (shRNAs) targeting GJB5, designated kdGJB5-sh1 and kdGJB5-sh2, were synthesized and sub-cloned into the GV369 lentiviral vector obtained from Genechem (Shanghai, China). Lentiviral particles were generated by transfecting these constructs, along with requisite viral packaging plasmids, into HEK-293 cells. Subsequently, the resulting lentiviral particles were employed to transduce the described cervical cancer cells or epithelial cells. Following selection (with puromycin), stable cells exhibiting GJB5 knockdown were established, with GJB5 expression validated through qPCR and Western blotting analysis.

### *GJB5* gene knockout

The primary cervical cancer cells were cultured in complete growth medium supplemented with polybrene until they reached 60% confluence. Subsequently, they were transduced with a lentiviral vector expressing Cas9 nuclease, kindly provided by Dr. Cao [29], to generate stable cells. These cells were then subjected to transduction with two distinct CRISPR/Cas9 constructs, each engineered to mediate GJB5 gene knockout and harboring unique single-guide RNA (sgRNA) sequences: koGJB5-sg1 and koGJB5-sg2. Following puromycin selection to enrich for transduced cells, single-cell clones were isolated by plating the cells in 96-well plates. The successful disruption of GJB5 gene expression was subsequently validated through DNA sequencing and Western blotting analysis. Control cells were generated by transducing cells with a lentiviral vector expressing Cas9 nuclease and a non-targeting scrambled control sgRNA (“koC”), also obtained from Dr. Cao [29].

### GJB5 overexpression

GJB5 overexpression was achieved using the hGJB5-expressing lentiviral GV369 construct, sourced from Genechem. The production of lentivirus involved transfecting the construct with packaging plasmids into HEK-293 cells. Following this, the resulting viral particles were used to infect cultured cervical cancer cells, which transitioned to puromycin-containing medium after 48 h. Stable cells with GJB5 overexpression were established after five passages, with GJB5 overexpression validation performed using qPCR and Western blotting techniques.

### Gαi3 (G alpha inhibitory protein 3) silencing or overexpression

Silencing of Gαi3 was achieved using a lentiviral shRNA (shGαi3), while overexpression was accomplished with a lentiviral construct (oeGαi3), both provided by Dr. Cao [29, 31, 32]. The stable cell selection procedure for these manipulations has been previously described [29, 31, 32].

### CCK-8 assay

Cell viability was evaluated utilizing the CCK-8 method. In detail, 3000 cells per well were placed into a 96-well plate and maintained for 96 h. Following this, 20 μL of CCK-8 solution was introduced to each well and incubated for 80 min. Viability was determined by measuring the absorbance at 440 nm with a microplate reader, indicating the reduction of the CCK-8 reagent.

### Colony formation assay

In brief, 13,000 cervical cancer cells per dish, subjected to specific genetic modifications, were seeded in 10-cm culture dishes. After a 10-day incubation period, colonies were stained and counted manually.

### Nuclear EdU/TUNEL staining

Cervical cancer or epithelial cells were cultured in 12-well plates for predetermined times. They were fixed using paraformaldehyde and permeabilized with Triton X-100. Staining was performed using TUNEL/EdU and DAPI dyes. Stained cells were imaged with a Zeiss fluorescence microscope (Oberkochen, Germany). The ratio of EdU/TUNEL-positive cells to DAPI-stained nuclei was assessed.

### Transwell assay

The specific cervical cancer cells, at a density of 8000 cells per condition, were placed on the upper side of Transwell inserts to migrate over 24 h. Cells that traversed to the lower surface were fixed and stained with crystal violet. For in vitro cell invasion assays, Matrigel was coated to the inserts.

### JC-1 staining

The mitochondrial membrane potential (MMP) was evaluated using a JC-1 assay kit from Invitrogen, following the manufacturer’s guidelines. The cervical cancer cells subjected to genetic modifications were incubated with JC-1 dye (3.5 μg/mL) for 25 min, and excess dye was removed through washing with warm PBS. The resulting green fluorescence, indicating reduced MMP, was measured at an excitation wavelength of 480 nm using fluorescence microscopy.

### Cytosolic Cytochrome c detection

Levels of Cytochrome c were measured via an enzyme-linked immunosorbent assay (ELISA) provided by Thermo-Fisher Invitrogen. This assay involves immobilizing specific capture antibodies against Cytochrome c on a microplate. Cytosolic protein lysates (0.8 μg/μL, 24 μg per sample) were added, allowing Cytochrome c to bind to the capture antibodies. A detection antibody linked to an enzyme was then introduced, forming a complex with the captured Cytochrome c. A substrate solution was added, resulting in an enzyme-driven colorimetric reaction, detected at 450 nm. A standard curve was created to determine Cytochrome c levels based on absorbance values obtained.

### Caspase-3 and Caspase-9 activity assessment

The activities of Caspase-3 and Caspase-9 were evaluated utilizing colorimetric assay kits (Thermo-Fisher Invitrogen), following the protocols provided by the manufacturer. In summary, cell or tissue lysates were prepared at a concentration of 0.8 μg/μL, with 24 μg allocated for each sample. These lysates were then incubated with specific substrates designed for caspases. The enzymatic cleavage of these substrates resulted in the formation of a colorimetric product, which was subsequently measured spectrophotometrically at a wavelength of 400 nm. A standard curve was consistently generated to accurately determine caspase activity.

### Constitutively-active mutant Akt1 (caAkt1)

Specifically, the lentivirus expressing the caAkt1 (S473D) variant, as previously documented (provided by Dr. Cao [29, 31, 32]), was introduced to cultured primary human cervical cancer cells. Following this introduction, stable cells expressing caAkt1 were established through selection with puromycin, with its expression validated through Western blotting assays.

### Xenograft assays

Female nude BALB/c mice, weighing between 17.7 and 18.2 grams, were obtained from Shanghai SLAC Laboratory Animal Co. (Shanghai, China). A total of seven million genetically modified primary cervical cancer cells were subcutaneously injected into the flanks of the mice. Tumor volumes, body weights, and daily growth rates (measured in mm³ per day) were recorded 18 days after cell inoculation in accordance with established guidelines [33]. TUNEL staining was subsequently performed on tumor sections using a Biyuntian kit (Wuxi, China), followed by washing and counterstaining with DAPI to visualize cell nuclei. All animal procedures were approved by the Institutional Animal Care and Use Committee (IACUC) and the Ethics Review Board of The Affiliated Kunshan Hospital of Jiangsu University.

### Statistical analysis

All in vitro experiments were conducted with five biological replicates. Data exhibiting normal distribution are presented as mean ± standard deviation (SD). Unpaired Student’s *t*-tests were employed to compare data between two experimental groups. For comparisons among three or more groups, one-way analysis of variance (ANOVA) was conducted, followed by post-hoc multiple comparison tests (Scheffé's and Tukey’s method). Statistical significance was considered at a *P*-value threshold of less than 0.05.

## Results

### *GJB5* expression and clinical outcomes in cervical cancer

**Fig. 1:**
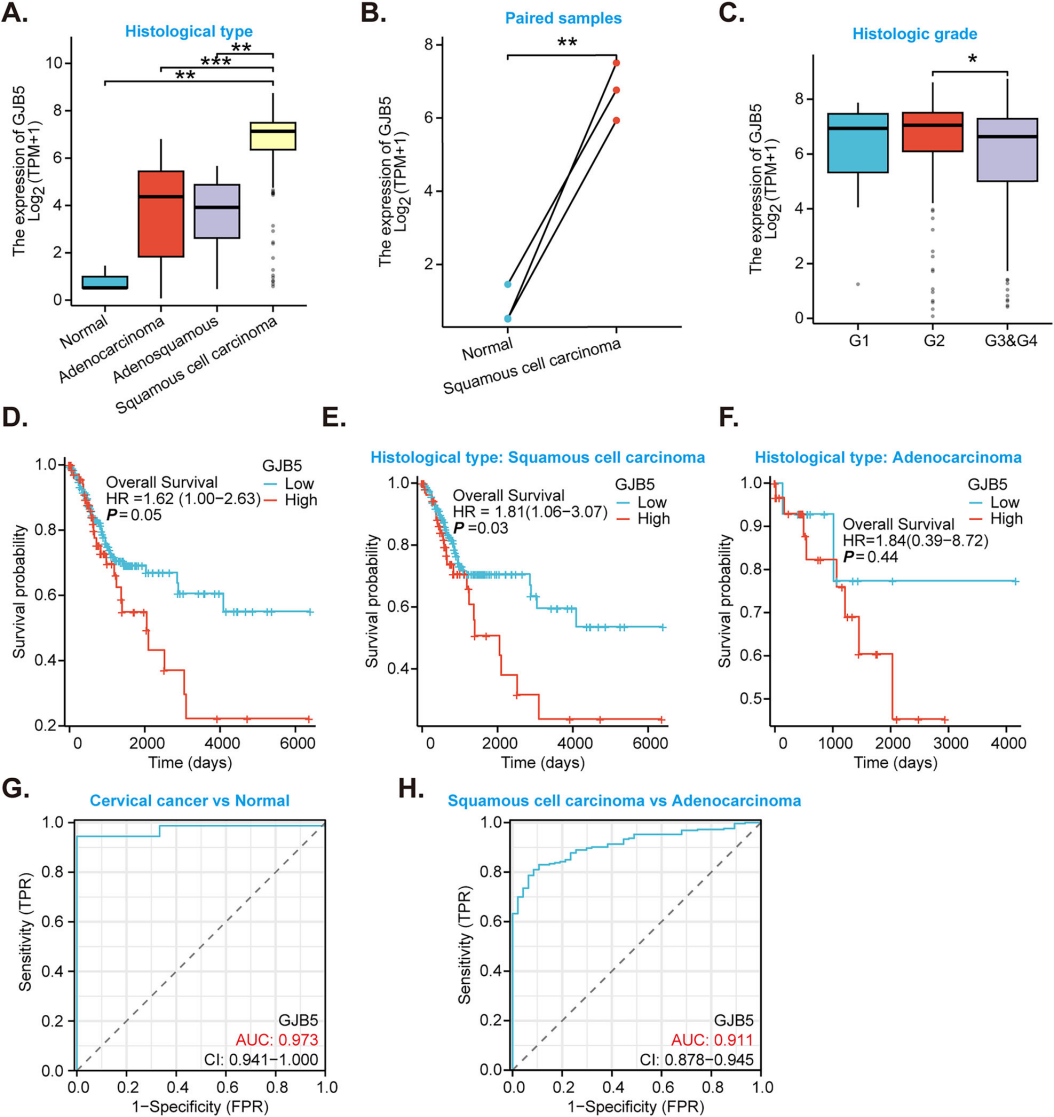
*GJB5* expression and clinical outcomes in cervical cancer. Box plot showing *GJB5* mRNA expression levels in normal tissues, adenocarcinoma, squamous cell carcinoma, and adenosquamous carcinoma tissues (**A**). Box plot comparing *GJB5* mRNA expression in paired normal and squamous cell carcinoma tissue samples (**B**). Box plot showing *GJB5* expression levels in G1, G2, and G3&G4 cervical cancer tumors (**C**). Kaplan–Meier survival curves for patients with high and low *GJB5* expression in cervical cancer patients (**D**), squamous cell carcinoma patients (**E**) and adenocarcinoma patients (**F**). Receiver Operating Characteristic (ROC) curve for *GJB5* mRNA expression in differentiating cervical cancer from normal tissue samples (**G**). ROC curve for *GJB5* mRNA expression in differentiating squamous cell carcinoma from adenocarcinoma (**H**). Error bars represent standard deviation. “TPM” stands for transcripts per million. “AUC” stands for area under curve. “CI” stands for confidence interval. “HR” stands for hazard rate. *“TPR”* stands for true positive rate. *“FPR”* stands for false positive rate. Data are presented as mean ± standard deviation (SD). *P < 0.05. **P < 0. 01. ***P < 0.001.

Gene expression analysis of cervical cancer specimens from The Cancer Genome Atlas (TCGA) revealed significant dysregulation of *GJB5* expression. Compared to normal cervical tissue (“Normal”), *GJB5* expression was markedly elevated in adenocarcinoma, squamous cell carcinoma (SCC), and adenosquamous carcinoma (Fig. 1A). This upregulation was further corroborated by paired sample analysis, which demonstrated a significant increase in *GJB5* expression in squamous cell carcinoma compared to normal tissues from the same patients (Fig. 1B). With respect to histological grade, G2 tumors displayed the highest levels of *GJB5* expression (Fig. 1C). To investigate the clinical significance of *GJB5* expression, Kaplan-Meier survival analysis was conducted. Results demonstrated a significant association between high *GJB5* overexpression and reduced overall survival in cervical cancer patients (Fig. 1D). This adverse prognostic impact of elevated *GJB5* expression was particularly evident in patients with squamous cell carcinoma (Fig. 1E). In contrast, no significant correlation was observed between *GJB5* expression and overall survival in adenocarcinoma patients (Fig. 1F).

We also studied other GJB family proteins whose expression/roles in cervical cancer had not been previously reported, including GJB3 and GJB4. Although analysis of TCGA data indicates upregulated expression of *GJB3* and *GJB4* mRNA in cervical cancer (Fig. S2A, B), their overexpression is not significantly associated with poorer patient prognosis (Fig. S2C, D).

To assess the diagnostic potential of *GJB5* expression, receiver operating characteristic (ROC) curve analysis was performed. Figure 1G demonstrated excellent discriminatory power for *GJB5* in distinguishing cervical cancer tissues from normal tissues, with an area under the curve (AUC) of 0.973. Furthermore, Fig. 1H revealed that *GJB5* expression can effectively differentiate between squamous cell carcinoma and adenocarcinoma, with an AUC of 0.911. These findings collectively suggest that *GJB5* expression may serve as a valuable biomarker for the diagnosis and subtyping of cervical cancer.

### The single-cell RNA sequencing demonstrates overexpression of *GJB5* in the epithelial population of cervical squamous cell carcinoma

**Fig. 2:**
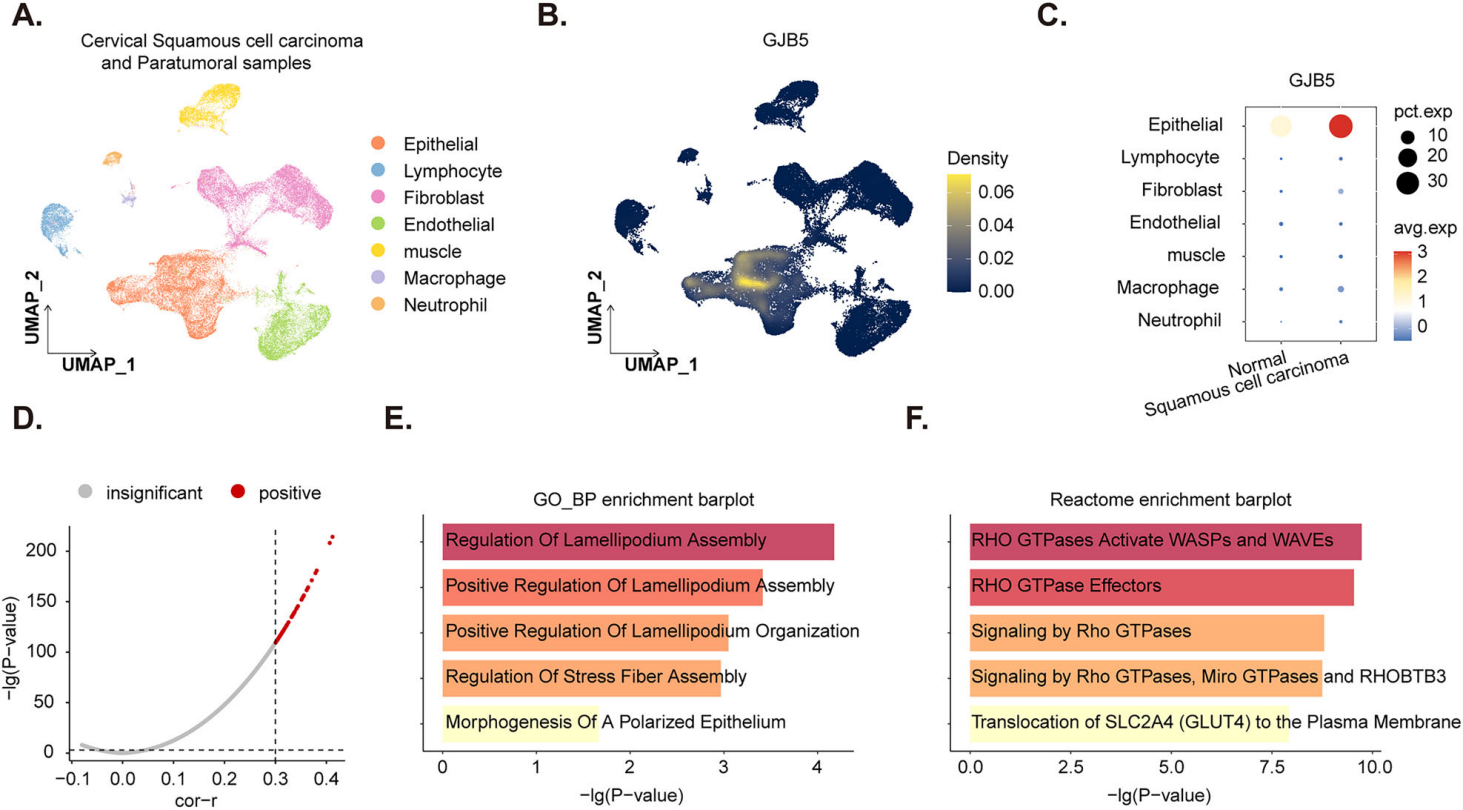
The single-cell RNA sequencing demonstrates overexpression of *GJB5* in the epithelial population of cervical squamous cell carcinoma. UMAP dimensionality reduction of single-cell RNA-seq data from cervical squamous cell carcinoma samples, showing distinct cell clusters (**A**). Density plot showing the expression of *GJB5* mRNA across different cell types, demonstrating its enrichment in epithelial cells (**B**). Comparison of *GJB5* expression in epithelial cells from cervical squamous cell carcinoma and paracancer normal tissues (**C**). Volcano plot showing the correlation between *GJB5* expression and other genes within the epithelial cell cluster (**D**). Genes with a correlation coefficient >0.3 and *P*-value < 0.05 are highlighted (**D**). Gene ontology (GO) enrichment analysis of genes highly correlated with *GJB5*, showing enrichment in biological processes related to cell motility and morphology (**E**). Reactome pathway enrichment analysis of genes highly correlated with *GJB5*, revealing significant enrichment in Rho GTPase signaling pathways (**F**).

To gain a deeper understanding of the cellular heterogeneity and gene expression dynamics in cervical cancer, we evaluated the cervical squamous carcinoma single-cell RNA sequencing data from the ArrayExpress database with the accession number E-MTAB-11948. This approach allows for the identification of cell-type-specific gene expression patterns. The results revealed a distinct expression pattern of *GJB5* in cervical squamous cell carcinoma. UMAP (uniform manifold approximation and projection) dimensionality reduction of the dataset identified distinct cell clusters corresponding to various cell types, including epithelial, lymphocyte, fibroblast, endothelial, muscle, macrophage, and neutrophil cells (Fig. 2A). The expression of *GJB5* was predominantly localized to epithelial cells, as shown by the density plot (Fig. 2B, C). Further analysis revealed a significant increase in *GJB5* expression in epithelial cells from cervical squamous cell carcinoma samples compared to paracancer normal tissue (Fig. 2B, C).

To explore the potential functional implications of *GJB5* upregulation in cervical squamous cell carcinoma, we performed correlation analysis of *GJB5* expression with all other genes within the epithelial cell cluster. Genes exhibiting a correlation coefficient greater than 0.3 and a *P*-value less than 0.05 were considered highly correlated with *GJB5* (Fig. 2D). Gene ontology (GO) enrichment analysis of these genes revealed significant enrichment in biological processes related to lamellipodium assembly, regulation of stress fiber assembly, and morphogenesis of a polarized epithelium (Fig. 2E). Additionally, pathway enrichment analysis using Reactome identified significant enrichment in pathways involving Rho GTPases, which regulate actin cytoskeleton dynamics (Fig. 2F). Thus, the scRNA-seq analysis demonstrates overexpression of *GJB5* in the epithelial population of cervical squamous cell carcinoma, suggesting its potential involvement in tumor progression and highlighting its potential as a therapeutic target.

### GJB5 overexpression in cervical cancer tissues from locally treated patients and different cervical cancer cells

**Fig. 3:**
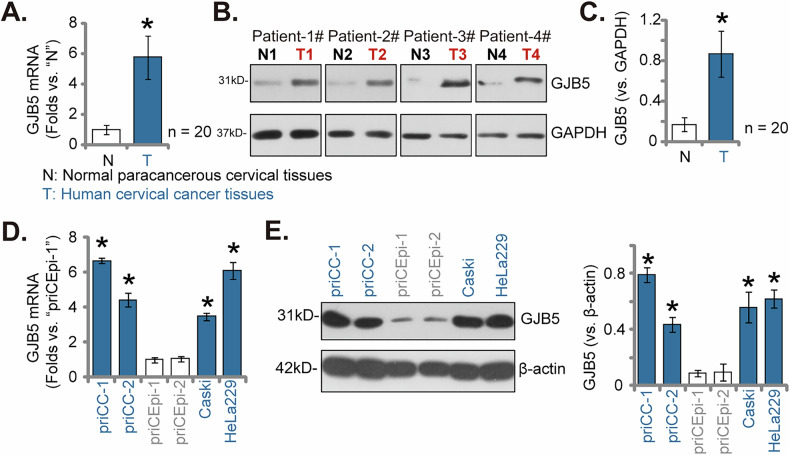
GJB5 overexpression in cervical cancer tissues from locally-treated patients and different cervical cancer cells. The expression levels of the listed genes and proteins in cervical cancer tissues (“T”) and matched normal paracancerous cervical epithelial tissues (“N”) were evaluated in a cohort of twenty (*n* = 20) primary cervical cancer patients, with results quantified (**A**–**C**). Additionally, *GJB5* mRNA and protein expression were assessed in the specified cervical cancer cells and cervical epithelial cells (**D**, **E**). Data are presented as mean ± standard deviation (SD) with *n* = 5 replicates. Asterisks (*) indicate statistically significant differences (*P* < 0.05) compared to the “N” tissues or priCEpi-1 cells. The experiments were independently replicated five times with consistent results.

Next, our study revealed a significant upregulation of GJB5 in cervical cancer tissues of locally-treated patients. Analysis of cervical cancer tissues from twenty (*n* = 20) patients (as reported previously [29]) indicated a substantial increase in *GJB5* mRNA levels when compared to matched paracancerous tissues (Fig. 3A). This elevated mRNA expression was further confirmed at the protein level. Western blotting analysis conducted on four representative patients (Patient-1# to Patient-4#) demonstrated markedly heightened GJB5 protein expression in cervical cancer tissues relative to their paracancerous normal counterparts (Fig. 3B). Comprehensive analysis of all 20 patient samples reinforced the significant upregulation of GJB5 protein in cervical cancer tissues (Fig. 3C).

To further investigate GJB5 expression in cervical cancer cells, we examined both primary and immortalized cells. Our results indicated that both *GJB5* mRNA (Fig. 3D) and protein (Fig. 3E) levels were significantly elevated in primary human cervical cancer cells (priCC-1 and priCC-2) and immortalized cervical cancer cell lines (Caski and HeLa229) compared to primary cervical epithelial cells (priCEpi-1 and priCEpi-2). Collectively, these findings underscore a consistent and significant overexpression of GJB5 in cervical cancer tissues and cells, suggesting its potential role in the pathogenesis of this disease.

### GJB5 silencing inhibits malignant behaviors in cervical cancer cells

**Fig. 4:**
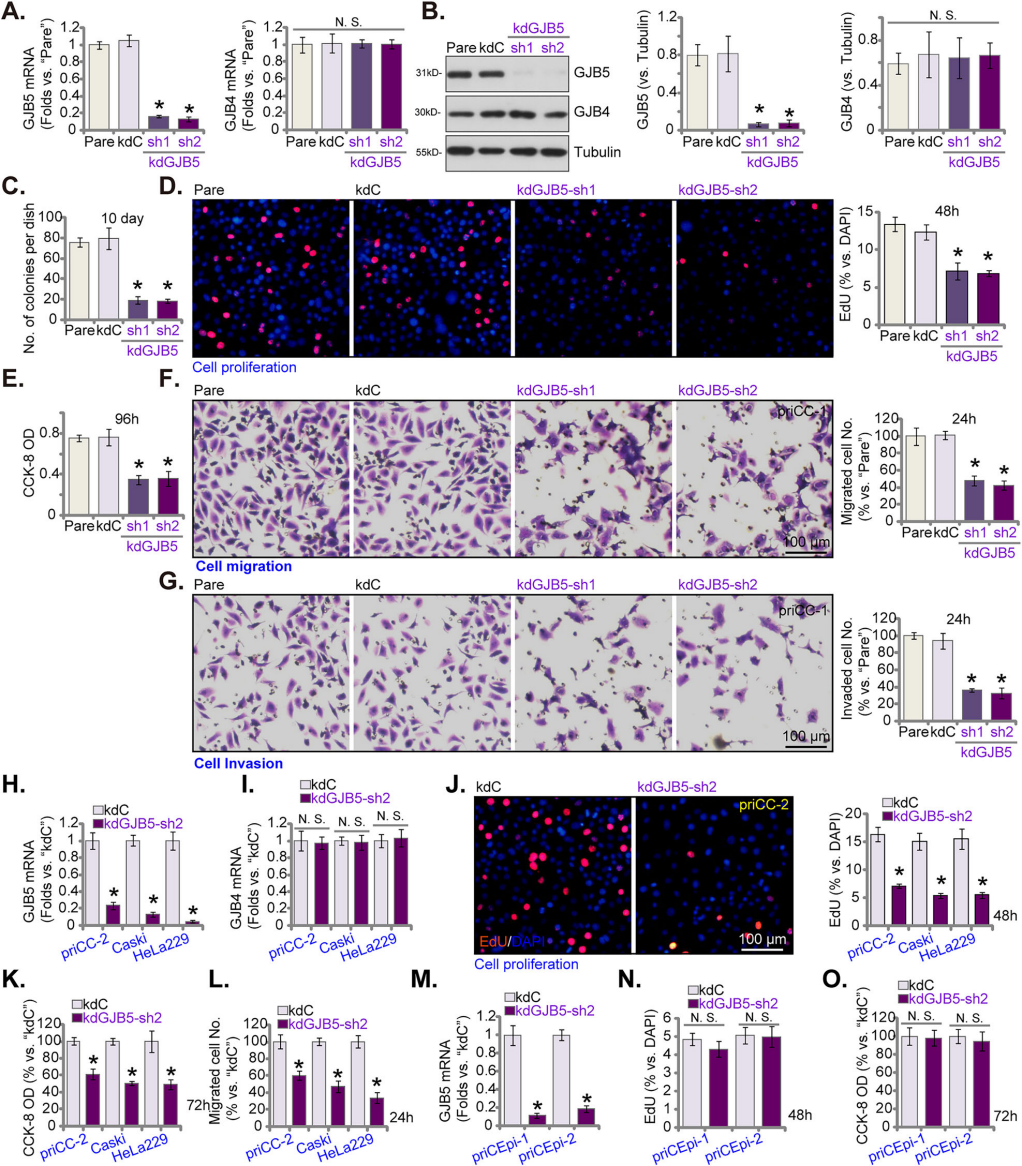
GJB5 silencing inhibits malignant behaviors in cervical cancer cells. qPCR (**A**) and western blotting (**B**) analysis showing GJB5/GJB4 mRNA and protein expression levels in priCC-1 primary human cervical cancer cells transfected with control shRNA (“kdC”) or GJB5-specific shRNA (kdGJB5-sh1 and kdGJB5-sh2). Cells were subsequently cultured in complete medium for designated time periods to assess colony formation (**C**), cell proliferation (by measuring the ratio of EdU-positive nuclei, **D**) and cell viability (via CCK-8 OD, **E**), as well as in vitro cell migration (using “Transwell” assays, **F**) and invasion (through “Matrigel Transwell” assays, **G**). Additionally, other patient-derived primary human cervical cancer cells (priCC-2) (**H**–**L**), along with the established Caski and HeLa229 cell lines (**H**–**L**), and primary human cervical epithelial cells (priCEpi-1 and priCEpi-2, derived from two patients) (**M**–**O**), were transduced with kdGJB5-sh2 or kdC. Stable cells were established following puromycin selection. The expression levels of specific mRNAs were evaluated (**H,** **I**, **M**). Cells were again cultured in complete medium for the specified time periods, and measurements for cell proliferation (**J**, **N**), cell viability (CCK-8 OD, **K**, **O**), and in vitro cell migration (**L**) were performed. “Pare” stands for parental control cells. Data are presented as mean ± standard deviation (SD) with *n* = 5 replicates. Asterisks (*) indicate statistically significant differences (*P* < 0.05) compared to the “kdC” treatment. “N.S.” denotes no statistically significant difference (*P* > 0.05). The experiments were independently replicated five times with consistent results. Scale bar represents 100 μm.

To investigate the possible functional role of GJB5 in cervical cancer malignant behaviors, we utilized lentiviral shRNA to knockdown (kd) GJB5 expression in priCC-1 primary human cervical cancer cells. Two distinct shRNA sequences (kdGJB5-sh1 and kdGJB5-sh2) were employed to achieve robust silencing. qPCR analysis revealed a significant decrease in *GJB5* mRNA expression in priCC-1 cells transfected with both shRNA constructs compared to control cells with scramble control shRNA (“kdC”) (Fig. 4A). Western blotting analysis also confirmed a significant reduction in GJB5 protein levels (Fig. 4B). The GJB4 and GJB5 genes encode closely related beta-connexin proteins that play roles in forming gap junctions and facilitating direct intercellular communication [34].The silencing of GJB5 did not affect the expression levels of GJB4 (Fig. 4A, B). Functional assays demonstrated a pronounced inhibitory effect of GJB5 silencing on key aspects of cervical cancer cell behaviors. Colony formation assays showed a significant reduction in the number of colonies formed by GJB5-silenced priCC-1 cells compared to control cells (Fig. 4C), indicating impaired growth potential. Furthermore, the nuclear EdU incorporation assays revealed a decrease in cell proliferation in GJB5-deficient cells (Fig. 4D). Consistent with these findings, CCK-8 assays demonstrated a significant reduction in cell viability in GJB5-silenced cells (Fig. 4E). To assess the impact of GJB5 on cell motility, we performed cell migration (“Transwell”) and invasion (“Matrigel Transwell”) assays. In both assays, GJB5-silenced priCC-1 cells exhibited significantly reduced migration and invasion capabilities compared to control cells (Fig. 4F, G). These findings collectively suggest that GJB5 plays a crucial role in promoting the growth, proliferation, survival and motility of cervical cancer cells.

To validate the role of GJB5 in multiple cervical cancer contexts, we expanded our investigation to additional cells, including the primary priCC-2 cervical cancer cells and the immortalized Caski and HeLa229 cell lines. Using the same kdGJB5-sh2 lentiviral construct, we observed consistent and robust downregulation of *GJB5* mRNA expression across all three cervical cancer cell types (Fig. 4H), while *GJB4* mRNA levels remained unaffected (Fig. 4I). This selective silencing of GJB5 led to a significant decrease in cell proliferation, as measured by nuclear EdU incorporation (Fig. 4J), and a reduction in cell viability, as assessed by CCK-8 assays (Fig. 4K). Furthermore, cell migration assays demonstrated a significant decrease in migratory capacity in GJB5-silenced cells compared to controls (Fig. 4L). To examine the impact of GJB5 on normal cervical epithelial cells, we utilized primary human cervical epithelial cells, priCEpi-1 and priCEpi-2. Similar to the cancer cells, *GJB5* mRNA expression was significantly downregulated in these cells following transfection with the kdGJB5-sh2 construct (Fig. 4M). However, in contrast to the cervical cancer cells, GJB5 silencing in primary epithelial cells did not result in a significant inhibition of cell proliferation (Fig. 4N) or a decrease in cell viability (Fig. 4O). The reason for the lack of effect in primary epithelial cells could be due to the relatively low expression of GJB5 in normal versus cancerous cells (see Figs. 1–3). Thus, these findings demonstrate that GJB5 plays an important role in promoting the survival, growth and proliferation of cervical cancer cells, while having limited impact on normal cervical epithelial cells.

### GJB5 silencing results in apoptosis activation in cervical cancer cells

**Fig. 5:**
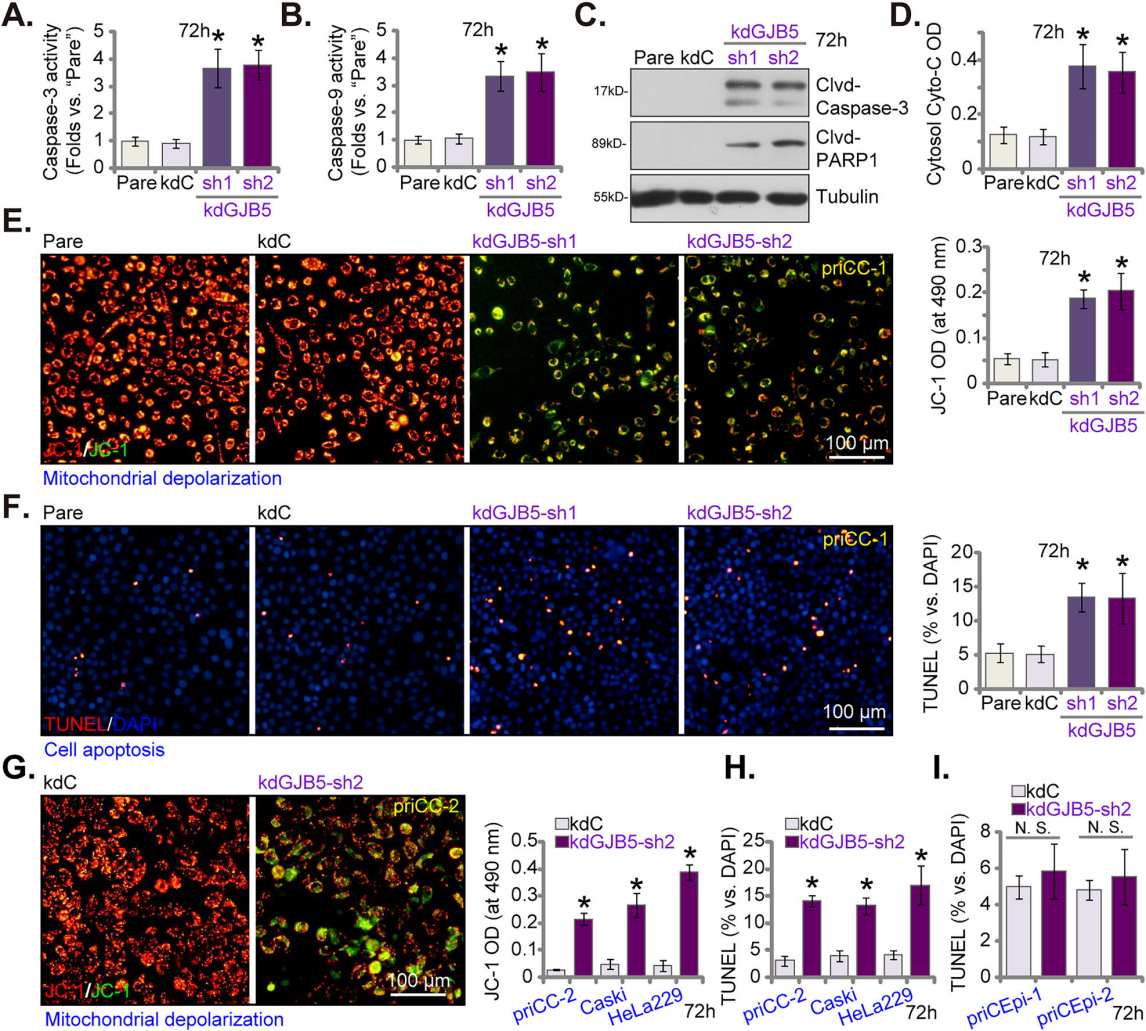
GJB5 silencing results in apoptosis activation in cervical cancer cells. The priCC-1 primary human cervical cancer cells with control shRNA (“kdC”) or GJB5-specific shRNA (kdGJB5-sh1 and kdGJB5-sh2) were cultured in complete medium for 72 h, and were subjected to Caspase-3 activity assay (**A**), Caspase-9 activity assay (**B**), Western blotting analysis of cleaved-Caspase-3 and cleaved-PARP1 (**C**), cytosol Cytochrome C release (**D**) and JC-1 staining for mitochondrial membrane potential (**E**). The nuclear TUNEL staining was carried out to detect DNA fragmentation (**F**). Additionally, other patient-derived primary human cervical cancer cells (priCC-2) (**G**, **H**), the established Caski and HeLa229 cell lines (**G**, **H**), and along with primary human cervical epithelial cells (priCEpi-1 and priCEpi-2, derived from two patients) (**I**) were transduced with kdGJB5-sh2 or kdC. Stable cells were established following puromycin selection. Cells were again cultured in complete medium for 72 h, and measurements include JC-1 staining for mitochondrial membrane potential (**G**) and TUNEL staining to detect DNA fragmentation (**H**, **I**). “Pare” stands for parental control cells. Data are presented as mean ± standard deviation (SD) with *n* = 5 replicates. Asterisks (*) indicate statistically significant differences (*P* < 0.05) compared to the “kdC” treatment. “N.S.” denotes no statistically significant difference (*P* > 0.05). The experiments were independently replicated five times with consistent results. Scale bar represents 100 μm.

To investigate the potential role of GJB5 on apoptosis, we again employed lentiviral shRNA to knockdown GJB5 expression in priCC-1 primary cervical cancer cells. Two distinct shRNA sequences (kdGJB5-sh1 and kdGJB5-sh2) were utilized to ensure the specificity of the observed effects (see Fig. 4). Our findings demonstrate that silencing GJB5 significantly induced apoptosis. We observed a significant increase in the activities of both Caspase-3 (Fig. 5A) and Caspase-9 (Fig. 5B), key executioners of apoptosis, in GJB5-silenced priCC-1 cells. This was further corroborated by the increased cleavage of Caspase-3 and its downstream target, PARP1 [Poly(ADP-ribose) polymerase 1], as detected by Western blotting analysis (Fig. 5C). Moreover, the levels of cytosol cytochrome C were significantly increased in GJB5 shRNA-expressing cells (Fig. 5D). In addition, JC-1 staining revealed a significant shift towards the green monomer form of JC-1, indicating a loss of mitochondrial membrane potential (MMP) (Fig. 5E). This depolarization is a hallmark of the intrinsic apoptotic pathway. The nuclear TUNEL staining demonstrated a significant increase in the number of cells with fragmented DNA, indicating apoptosis (Fig. 5F).

To further investigate the role of GJB5 in apoptosis, we extended our studies to additional cervical cancer cells, including primary priCC-2 cells and the immortalized Caski and HeLa229 lines. Using the same kdGJB5-sh2 lentiviral construct (see Fig. 4), we observed consistent results in these cancer cell types: silencing GJB5 expression resulted in mitochondrial depolarization, as indicated by a significant increase in the green monomer form of JC-1 (Fig. 5G), and increased apoptosis, as confirmed by a significant increase in the number of TUNEL-positive cells (Fig. 5H). In contrast, silencing GJB5 expression in primary human cervical epithelial cells (priCEpi-1 and priCEpi-2) using the same kdGJB5-sh2 construct (see Fig. 4) did not significantly induce apoptosis, as the TUNEL-positive nuclei ratio was not significantly changed (Fig. 5I). These findings suggest that the pro-apoptotic effect of GJB5 silencing is specific to cervical cancer cells.

### GJB5 knockout inhibits malignant behaviors in cervical cancer cells

**Fig. 6:**
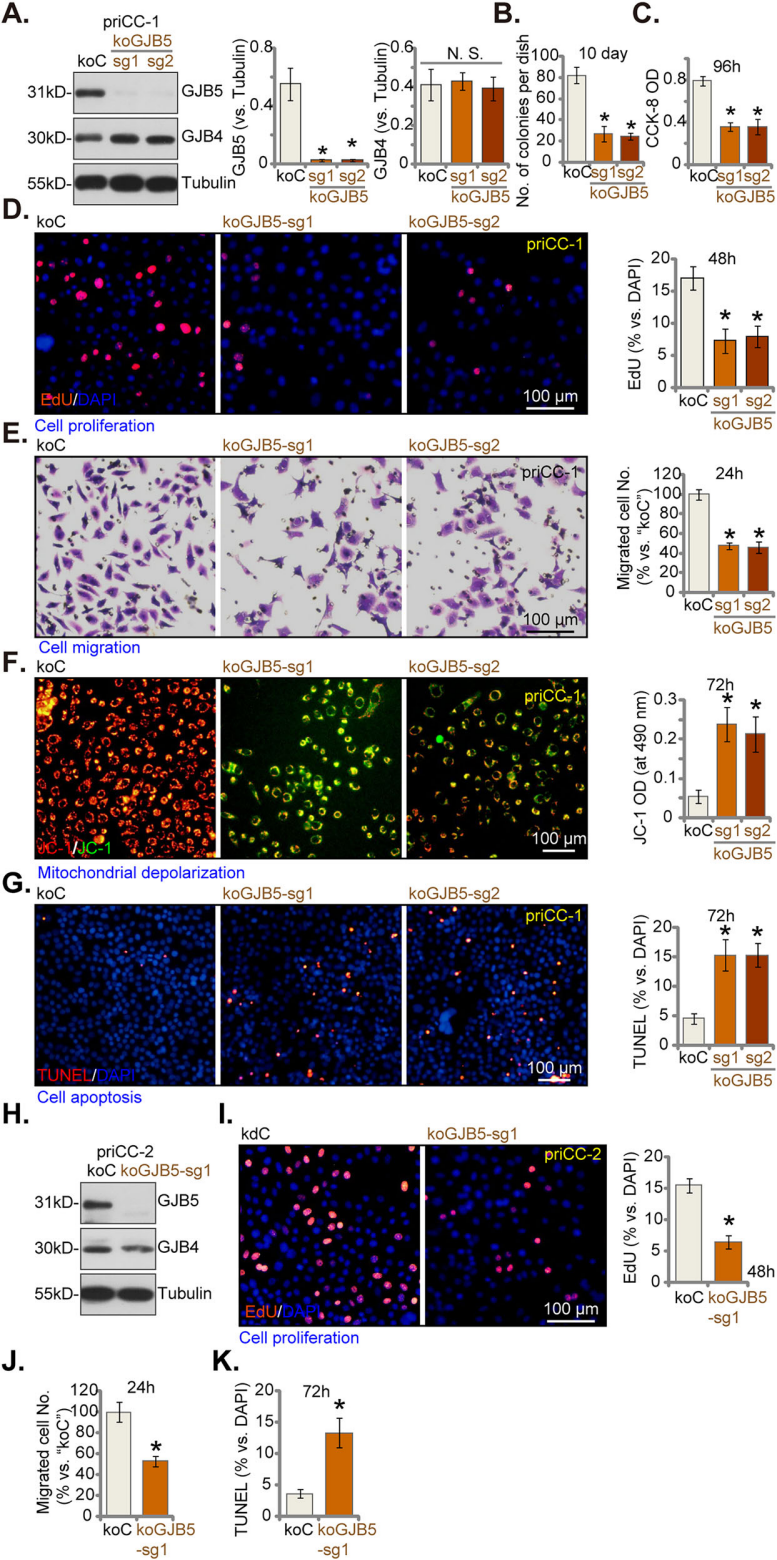
GJB5 knockout inhibits malignant behaviors in cervical cancer cells. The priCC-1 (**A**–**H**) or pri-CC-2 (**I**–**L**) primary human cervical cancer cells transfected with the lentiviral CRIPR/Cas9 construct containing control sgRNA (“koC”) or koGJB5-sg1/koGJB5-sg2 were established, the expression levels of listed proteins served were tested (**A**, **H**). Cells were subsequently cultured in complete medium for designated time periods to assess colony formation (**B**), cell viability (CCK-8 OD, **C**), cell proliferation (by measuring the ratio of EdU-positive nuclei, **D** and **I**), and in vitro cell migration (using “Transwell” assays, **E**, **J**). JC-1 staining for mitochondrial membrane potential (**F**) and nuclear TUNEL staining to detect cell apoptosis (**G**, **K**) were also carried out. Data are presented as mean ± standard deviation (SD) with n = 5 replicates. Asterisks (*) indicate statistically significant differences (*P* < 0.05) compared to the “koC” treatment. “N.S.” denotes no statistically significant difference (*P* > 0.05). The experiments were independently replicated five times with consistent results. Scale bar represents 100 μm.

To confirm the specificity of the observed effects of GJB5 silencing, we employed CRISPR/Cas9-mediated gene editing to generate GJB5 knockout (koGJB5) in priCC-1 cervical cancer cells. Two distinct single guide RNA (sgRNA) sequences (koGJB5-sg1 and koGJB5-sg2) were utilized to target GJB5, resulting in robust depletion of GJB5 protein without affecting the expression of GJB4 (Fig. 6A). GJB5 KO significantly impacted malignant behaviors in priCC-1 cells. Colony formation assays demonstrated a significant decrease in the number of colonies formed by koGJB5 cells compared to control cells (koC), indicating reduced cell proliferation (Fig. 6B). Cell viability, tested via CCK-8 assay, was also decreased (Fig. 6C). This was further supported by EdU incorporation assays, which showed a significant reduction in DNA synthesis in koGJB5 cells (Fig. 6D). Additionally, “Transwell” assays revealed a significant reduction in cell migration capacity in koGJB5 cells (Fig. 6E). koGJB5 cells also exhibited a significant increase in the green monomer form of JC-1, indicating mitochondrial depolarization (Fig. 6F), and increased apoptosis, as confirmed by a significant increase in the number of TUNEL-positive cells (Fig. 6G).

CRISPR/Cas9-mediated gene editing was extended to priCC-2 primary cervical cancer cells using the koGJB5-sg1 construct. Western blotting analysis confirmed efficient depletion of GJB5 protein in priCC-2 cells transfected with koGJB5-sg1 (Fig. 6H). Consistent with the findings in priCC-1 cells, GJB5 knockout in priCC-2 cells led to a significant decrease in cell proliferation, as evidenced by reduced EdU incorporation (Fig. 6I). Furthermore, “Transwell” assay results revealed a significant impairment in cell migration capacity in koGJB5-sg1-transfected priCC-2 cells (Fig. 6J). TUNEL staining demonstrated a significant increase in the number of cells with positive TUNEL staining after koGJB5-sg1 treatment, indicating apoptosis activation (Fig. 6K). These findings, in conjunction with the shRNA-mediated knockdown experiments, strongly suggest that GJB5 plays a critical role in regulating malignant cellular functions in cervical cancer cells.

### GJB5 overexpression promotes malignant behaviors in cervical cancer cells

**Fig. 7:**
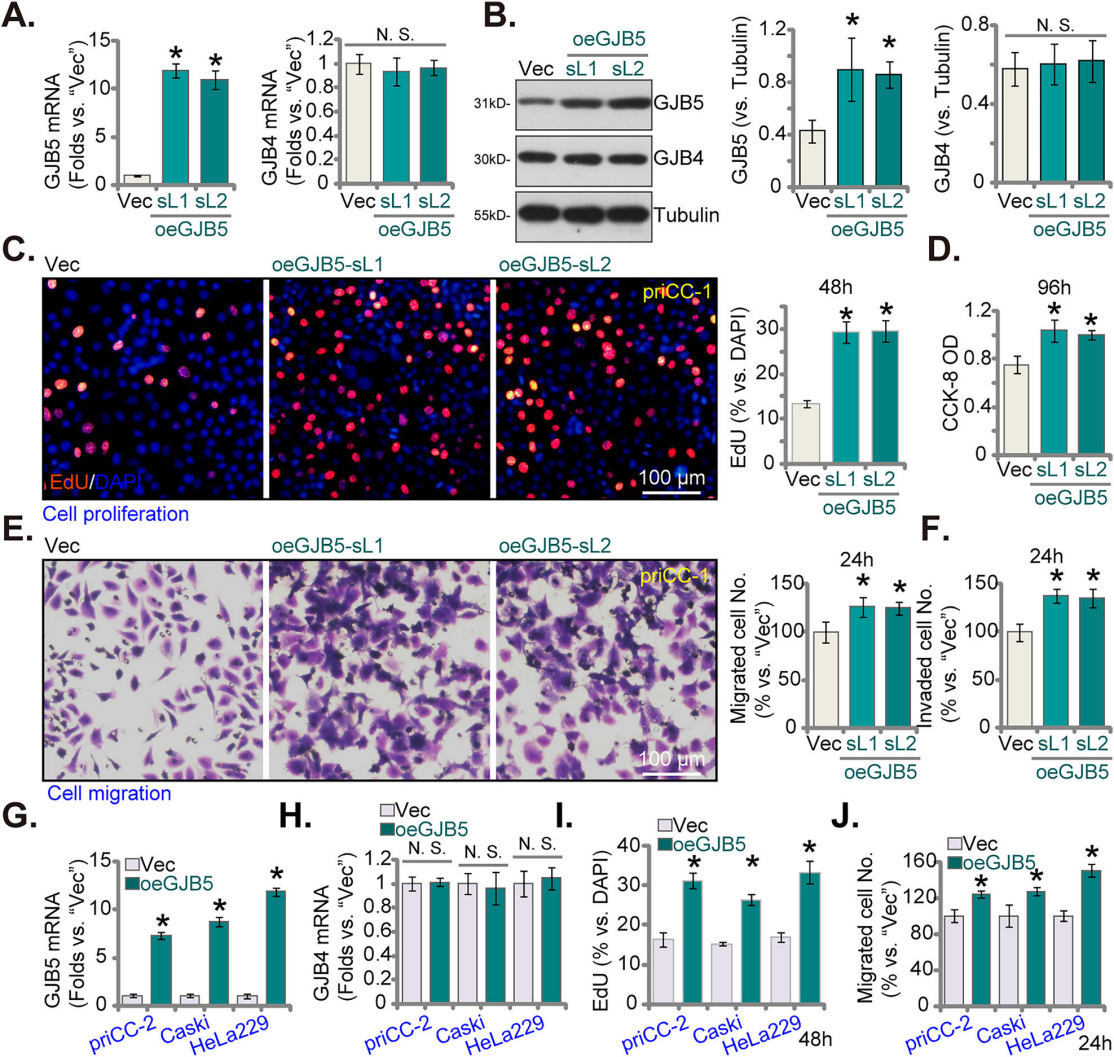
GJB5 overexpression promotes malignant behaviors in cervical cancer cells. *GJB5* and *GJB4* mRNA expression levels in priCC-1 cells transduced with the control vector (“Vec”) or the lentiviral GJB5-expressing construct (oeGJB5-sL1 or oeGJB5-sL2, two stable cell selections) were tested via quantitative PCR analysis (**A**), with Western blotting analysis of GJB5 and GJB4 protein expression in these priCC-1 cells (**B**). Cells were subsequently cultured in complete medium for designated time periods to assess cell proliferation (by measuring the ratio of EdU-positive nuclei, **C**), cell viability (via CCK-8 OD, **D**), and in vitro cell migration (using “Transwell” assays, **E**) and invasion (through “Matrigel Transwell” assays, **F**). Additionally, other patient-derived primary human cervical cancer cells (priCC-2) along with the established Caski and HeLa229 cell lines were stably transduced with the lentiviral GJB5-expressing construct (oeGJB5) or control vector (“Vec”), GJB5 and GJB4 mRNA expression levels were tested (**G** and **H**); Cells were subsequently cultured in complete medium for designated time periods to assess cell proliferation (by measuring the ratio of EdU-positive nuclei, **I**) and migration (using “Transwell” assays, **J**). Data are presented as mean ± standard deviation (SD) with *n* = 5 replicates. Asterisks (*) indicate statistically significant differences (*P* < 0.05) compared to the “Vec” treatment. “N.S.” denotes no statistically significant difference (*P* > 0.05). The experiments were independently replicated five times with consistent results. Scale bar represents 100 μm.

Next, we propose that ectopic GJB5 overexpression might promote malignant behaviors of cervical cancer cells. Therefore, priCC-1 primary cells were transduced with a lentiviral vector expressing GJB5. Two stable cell selections with increased GJB5 expression, designated oeGJB5-sL1 and oeGJB5-sL2, were established. qPCR analysis confirmed a significant increase in *GJB5* mRNA levels in both oeGJB5-sL1 and oeGJB5-sL2 cells compared to the vector control (Vec) (Fig. 7A). Western blotting analysis further validated these findings, showing increased GJB5 protein expression in these cells (Fig. 7B). GJB5 overexpression did not significantly alter the expression levels of GJB4 in priCC-1 cells (Fig. 7A, B). GJB5 overexpression significantly promoted cell proliferation (nuclear EdU incorporation) in oeGJB5-sL1 and oeGJB5-sL2 cells (Fig. 7C). Moreover, CCK-8 cell viability was increased in GJB5-overexpressing cells (Fig. 7D). Further assays revealed a significant increase in cell migration and invasion capacities in oeGJB5-sL1 and oeGJB5-sL2 cells (Fig. 7E, F). Next, we transduced primary priCC-2 cells and the immortalized Caski and HeLa229 cell lines with the same lentiviral vector expressing GJB5. Stable cells, oeGJB5, were established. qPCR analysis confirmed a significant increase in *GJB5* mRNA levels in all three cell types compared to the vector control (Fig. 7G), leaving *GJB4* mRNA expression unchanged (Fig. 7H). EdU incorporation assays demonstrated a significant increase in DNA synthesis in oeGJB5 cells compared to the vector control in all three cell types (Fig. 7I). Moreover, Transwell migration assays revealed a significant increase in cell migration capacity in oeGJB5 cervical cancer cells (Fig. 7J). Thus, GJB5 overexpression promotes several key malignant behaviors in cervical cancer cells, including increased proliferation, migration, and invasion.

### GJB5 positively regulates the Akt-mTOR signaling pathway in cervical cancer cells

**Fig. 8:**
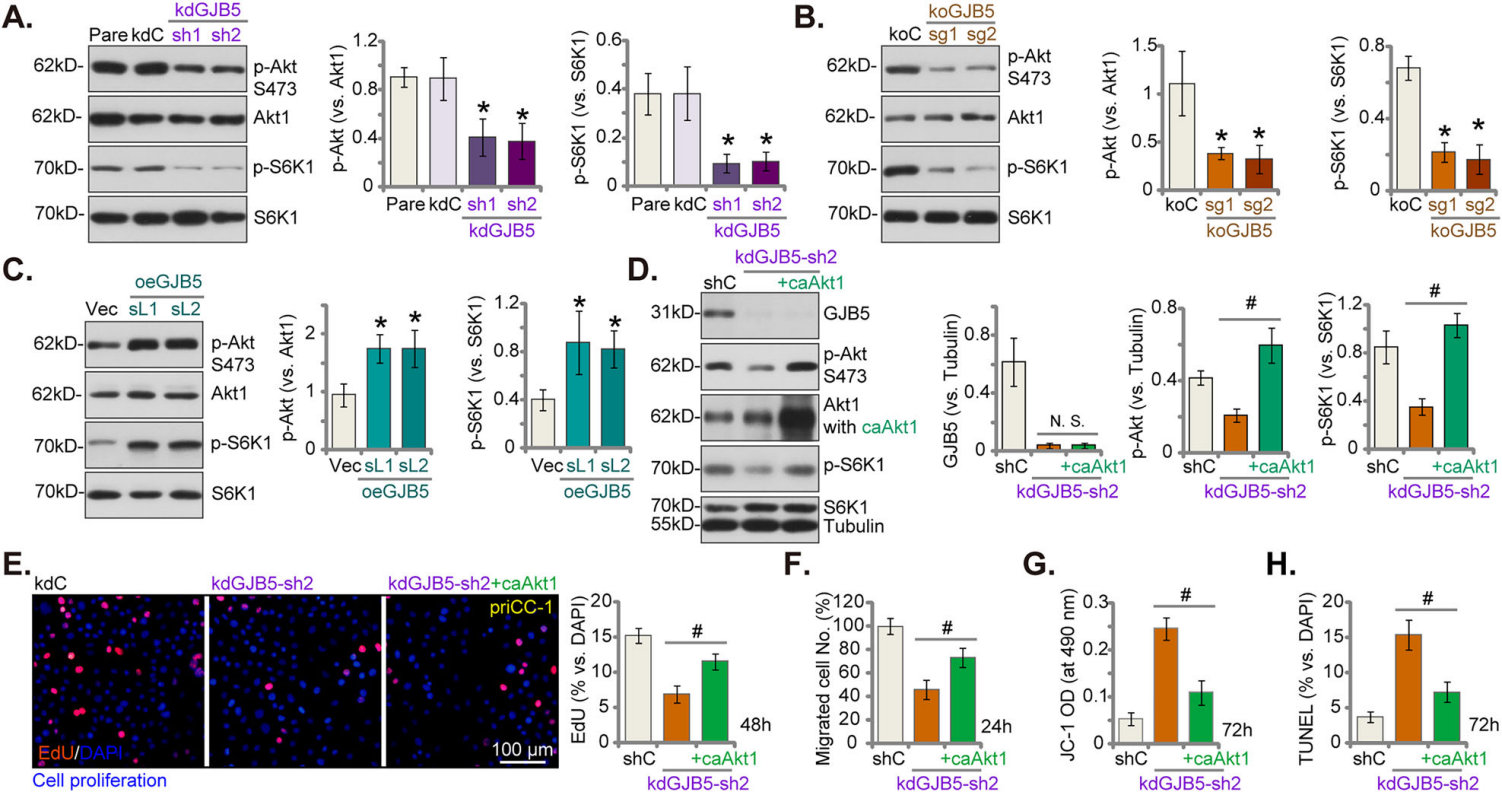
GJB5 positively regulates the Akt-mTOR signaling pathway in cervical cancer cells. Expression levels of the listed proteins in the priCC-1 primary human cervical cancer cells with the control shRNA (“kdC”), the GJB5-specific shRNA (kdGJB5-sh1, kdGJB5-sh2), the lentiviral CRIPR/Cas9 construct containing control sgRNA (“koC”), the lentiviral CRIPR/Cas9 construct expressing koGJB5-sg1 or koGJB5-sg2, the control vector (“Vec”) or the lentiviral GJB5-expressing construct (oeGJB5-sL1 or oeGJB5-sL2, two stable cell selections) were shown (**A**–**C**). The kdGJB5-sh2-expressing priCC-1 primary cancer cells were further transduced with or without a lentiviral constitutively-active Akt1 (caAkt1, S473D) mutant, with stable cells formed. Expression of listed proteins was shown (**D**). Cells were subsequently cultured in complete medium for designated time periods to assess cell proliferation (by measuring the ratio of EdU-positive nuclei, **E**) and in vitro cell migration (using “Transwell” assays, **F**); JC-1 staining for mitochondrial membrane potential (**G**) and nuclear TUNEL staining to detect cell apoptosis (**H**) were also carried out. Data are presented as mean ± standard deviation (SD) with *n* = 5 replicates. Asterisks (*) indicate statistically significant differences (*P* < 0.05) compared to the “kdC”/“koC”/“Vec” treatment. ^#^ indicates *P* < 0.05 (**D**–**H**). “N.S.” denotes no statistically significant difference (*P* > 0.05). The experiments were independently replicated five times with consistent results. Scale bar represents 100 μm.

The observation that GJB5 silencing or depletion exerts pleiotropic effects on cervical cancer cell behaviors, including inhibition of viability, proliferation, and migration, and enhancement of apoptosis (see Figs. 4–6), suggests that GJB5 may regulate key oncogenic signaling cascades [35–38]. Recognizing the Akt-mTOR pathway as a key driver of cervical cancer malignancy [37, 38], we focused our investigation on this pathway. In priCC-1 primary human cervical cancer cells, GJB5 silencing via shRNA (kdGJB5-sh1, kdGJB5-sh2, see Figs. 4 and 5) or CRISPR/Cas9-mediated knockout (koGJB5-sg1, koGJB5-sg2, see Fig. 6) significantly reduced phosphorylation of Akt (Ser-473) and its downstream target, S6K1, as determined by western blotting analysis (Fig. 8A, B). Conversely, GJB5 overexpression (oeGJB5-sL1, oeGJB5-sL2, see Fig. 7) augmented Akt-S6K1 phosphorylation (Fig. 8C). Expression levels of total Akt1 and S6K1 were unchanged following GJB5 depletion or overexpression in priCC-1 cells (Fig. 8A–C). These findings suggest that GJB5 positively regulates the Akt-mTOR signaling pathway in cervical cancer cells. To determine whether Akt inactivation mediates the anti-cervical cancer effects of GJB5 silencing, we introduced a constitutively-active Akt1 (caAkt1, S473D) mutant into GJB5-silenced priCC-1 cells, and it completely restored Akt-S6K1 phosphorylation without affecting GJB5 protein expression (Fig. 8D). Introduction of caAkt1 ameliorated the inhibitory effects of GJB5 silencing on priCC-1 cell proliferation (Fig. 8E) and migration (Fig. 8F). In addition, kdGJB5-sh2-induced mitochondrial depolarization (JC-1 green monomer accumulation, Fig. 8G) and apoptosis (Fig. 8H) were also inhibited with caAkt1 treatment in priCC-1 cells. These findings suggest that GJB5 plays an important role in promoting the Akt-mTOR signaling pathway in cervical cancer cells.

### GJB5 interacts with Gαi3 to promote Akt signaling activation in cervical cancer cells

**Fig. 9:**
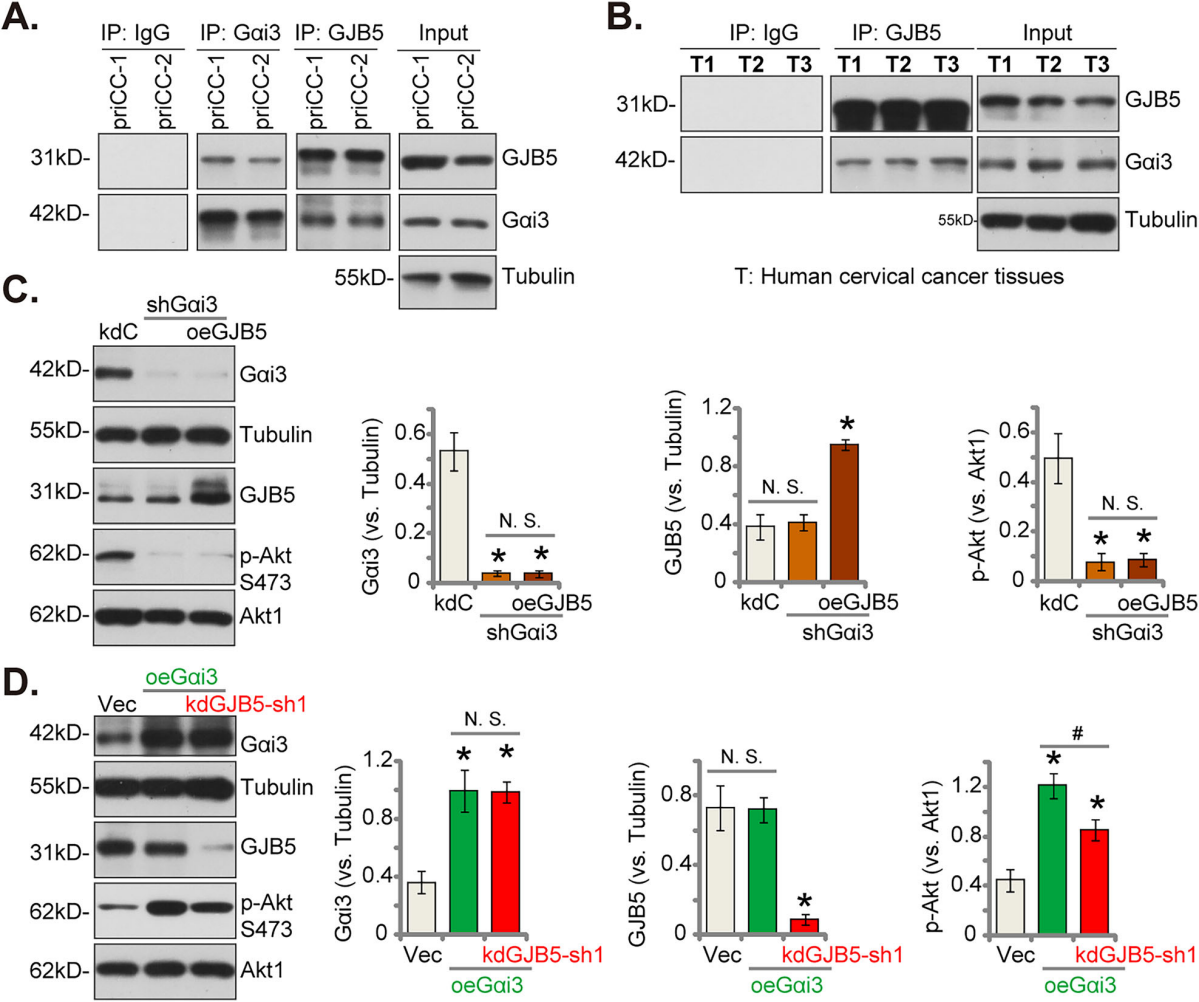
GJB5 interacts with Gαi3 to promote Akt signaling activation in cervical cancer cells. Co-immunoprecipitation (Co-IP) analysis showing interaction between GJB5 and Gαi3 proteins in priCC-1 and priCC-2 primary human cervical cancer cells (**A**) or in the listed human cervical cancer tissues (**B**). Western blotting analysis showing the effect of Gαi3 silencing (shGαi3), with or without GJB5 overexpression, on Akt phosphorylation in priCC-1 cells (**C**). Western blotting analysis showing the effect of Gαi3 overexpression, with or without GJB5 silencing (by kdGJB5-sh1), on Akt phosphorylation in priCC-1 cells (**D**). Data are presented as mean ± standard deviation (SD) with *n* = 5 replicates. Asterisks (*) indicate statistically significant differences (*P* < 0.05) compared to the “kdC”/“Vec” treatment. ^#^ indicates *P* < 0.05 (**D**). “N.S.” denotes no statistically significant difference (*P* > 0.05). The experiments were independently replicated five times with consistent results.

Results in Fig. 8 confirmed that GJB5 is important for Akt-mTOR cascade activation, we tested its possible underlying mechanism. A recent study has shown that Gαi3 protein is essential in regulating Akt-mTOR cascade activation in cervical cancer [29], possibly by associating with multiple receptor tyrosine kinases (RTKs) and non-RTK receptors [31, 32, 39–42]. We therefore explored GJB5’s potential interaction with Gαi3. Co-IP assays in priCC-1 and priCC-2 primary cervical cancer cells revealed a physical interaction between GJB5 and Gαi3 (Fig. 9A). This interaction was further validated in human cervical cancer tissues of three representative patients mentioned in Fig. 3 (Fig. 9B). To assess the functional significance of this interaction, we silenced Gαi3 using targeted shRNA (shGαi3) in priCC-1 cells. In line with the previous findings, Gαi3 silencing resulted in a significant decrease in Akt phosphorylation (Fig. 9C). Importantly, overexpression of GJB5 using the construct described in Fig. 7 failed to rescue Akt activation in Gαi3-silenced cells (Fig. 9C), indicating that Gαi3 is essential for GJB5-mediated Akt activation. Overexpression of Gαi3 in priCC-1 cells led to increased Akt phosphorylation in priCC-1 cells (Fig. 9D). Significantly, silencing GJB5 using kdGJB5-sh1 partially inhibited Akt activation induced by Gαi3 overexpression (Fig. 9D) in priCC-1 cells, suggesting that GJB5 is involved in Gαi3-mediated Akt activation. These findings demonstrate that GJB5 associates with Gαi3 and is required for Gαi3-mediated Akt activation in cervical cancer cells. This suggests a novel mechanism by which GJB5 contributes to Akt activation and cervical cancer progression.

### Silencing of GJB5 impedes cervical cancer xenograft growth in nude mice

**Fig. 10:**
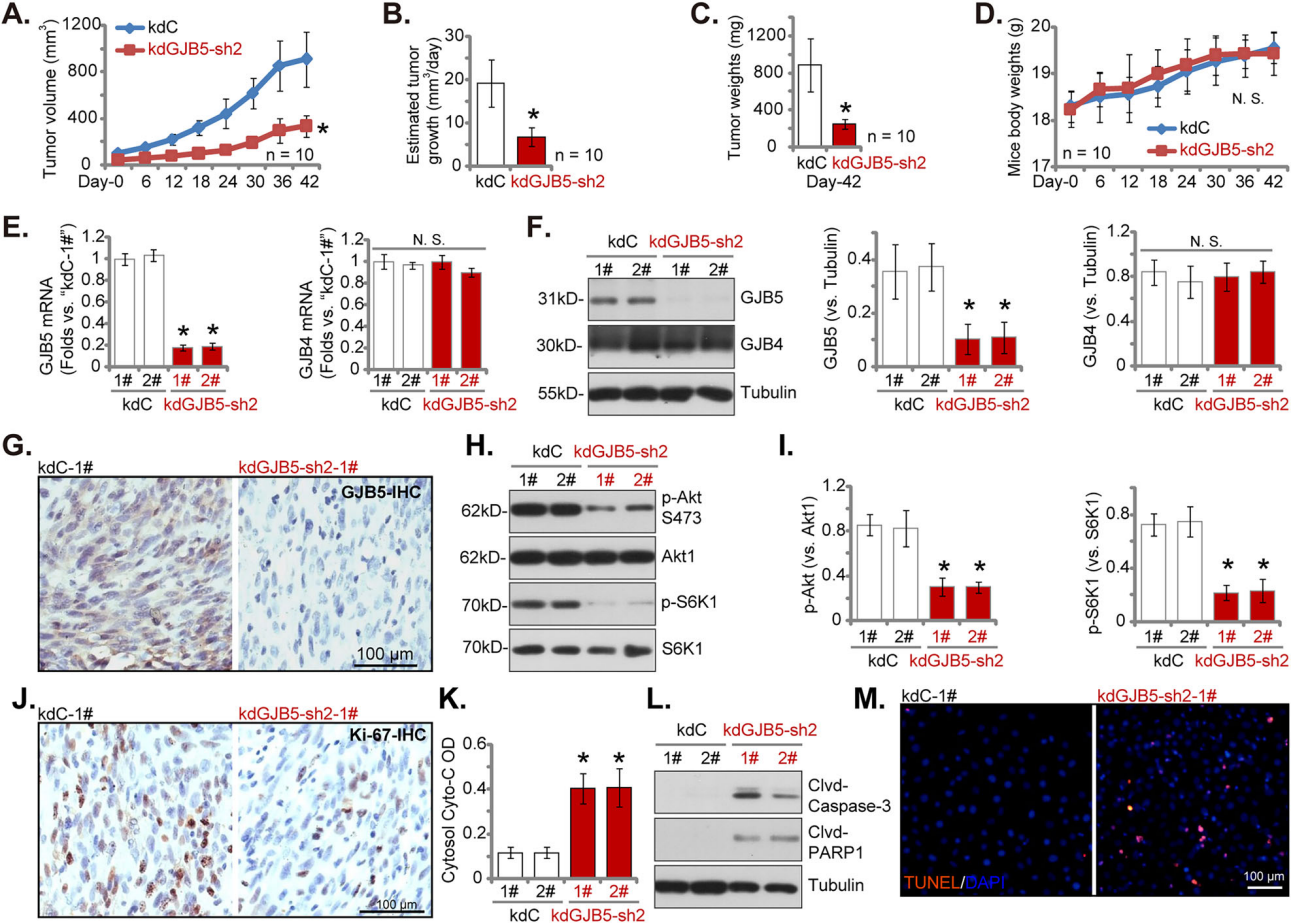
Silencing of GJB5 impedes cervical cancer xenograft growth in nude mice. The priCC-1 primary cervical cancer cells stably transfected with GJB5 shRNA (kdGJB5-sh2) or control shRNA (kdC) were subcutaneously injected into the flanks of female nude mice at a density of seven million cells per mouse. Ten mice were included in each experimental group (*n* = 10). Tumor growth was monitored for 42 days, with tumor volume (**A**, mm³) and mouse body weight (**D**, g) measured every six days starting from day 18 post injection, designated as “Day-0”. Daily tumor growth rates were calculated (**B**). At day 42, mice were euthanized, and tumors were excised and weighed (**C**). For the subsequent experiments, two xenografts from each group (labeled as 1# and 2#) were selected. Tissue lysates were prepared for the analysis of GJB5/4 expression levels (**E**, **F**), Akt and S6K1 phosphorylation/expression levels (**H**, **I**), and cleaved-caspase-3 and cleaved-PARP1 levels (**L**). Additionally, cytochrome C levels in the tissue lysates were assessed (**K**). Immunohistochemistry (IHC) was performed on tumor sections to assess GJB5 protein expression (**G**) and nuclear Ki-67 counts (**J**). Additionally, TUNEL/DAPI staining was performed in the xenograft sections to evaluate apoptosis (**M**). Data are presented as mean ± standard deviation (SD). Statistical significance was determined using a *P*-value of less than 0.05 (*P < 0.05) compared to the “kdC” group. “N.S.” indicates non-significant differences (*P* > 0.05). For analyses related to tumor volume (**A**), tumor growth rate (**B**), tumor weight (**C**), and mouse body weight (**D**), data were obtained from ten mice per group. For analyses of **E**–**M**, each xenograft was divided into five segments for individual analysis. A scale bar of 100 μm is provided.

To investigate the functional role of GJB5 in the in vivo growth of cervical cancer cells, we established subcutaneous xenograft models in nude mice using priCC-1 cells expressing either kdGJB5-sh2 or control (kdC) shRNA (see Figs. 4–6). Tumor volume was monitored 18 days after cell injection (labeled as “Day-0”) and over a 42-day period. A significant reduction in tumor growth was observed in the kdGJB5-sh2 group compared to the control group (Fig. 10A). Daily growth measurements further corroborated this finding, demonstrating a pronounced suppression of tumor expansion in the kdGJB5-sh2 xenografts (Fig. 10B). At the endpoint (“Day-42”), kdGJB5-sh2 xenografts exhibited significantly lower tumor weights compared to control xenografts (Fig. 10C). No significant changes in body weight were observed in either group throughout the experiment (Fig. 10D). To further elucidate the underlying mechanisms, we analyzed GJB5 expression in the harvested xenografts, with two xenografts acquired for each group. qPCR assays demonstrated a significant reduction in *GJB5* mRNA levels in kdGJB5-sh2 xenografts (Fig. 10E), along with a significant downregulation of GJB5 protein (Fig. 10F). GJB4 expression remained unchanged (Fig. 10E, F). IHC staining confirmed the efficient knockdown of GJB5 protein expression in kdGJB5-sh2 xenografts (Fig. 10G).

Given the potential involvement of the Akt-mTOR pathway in GJB5-mediated cervical cancer cell progression in vitro (see Figs. 8 and 9), we examined the activation status of this pathway. Western blotting analysis revealed a significant decrease in the phosphorylation levels of Akt and its downstream target, S6K1, in kdGJB5-sh2 xenografts compared to controls (Fig. 10H, I). A significant reduction in Ki-67-positive cells was observed in kdGJB5-sh2 xenografts compared to controls (Fig. 10J), indicating a significant suppression of cancer cell proliferation in vivo. We also observed evidence of increased apoptosis in kdGJB5-sh2 xenografts. Cytochrome C levels were elevated in the kdGJB5-sh2 xenograft tissues compared to controls (Fig. 10K). Western blotting analysis revealed increased levels of cleaved-caspase-3 and cleaved-PARP1 in kdGJB5-sh2 xenografts (Fig. 10L). TUNEL staining provided further evidence of apoptosis, showing a higher proportion of TUNEL-positive nuclei in kdGJB5-sh2 xenografts compared to controls (Fig. 10M). Collectively, these findings demonstrate that silencing of GJB5 led to the inhibition of the Akt-mTOR pathway, induction of apoptosis, and suppression of proliferation in priCC-1 xenografts in vivo.

## Discussion

Current therapeutic targets have made significant contributions to the management of cervical cancer, yet the limitations of existing treatments underscore the critical need for exploring novel targets [9, 10, 12, 14, 17]. Despite advancements in immunotherapy and other targeted therapies, the heterogeneity of cervical cancer and the development of resistance to standard treatments highlight the urgency of identifying alternative molecular pathways [9, 10, 12, 14, 17]. Novel therapeutic targets can enhance treatment efficacy, reduce side effects, and improve patient outcomes by addressing the underlying mechanisms driving tumor progression and metastasis [9, 10, 12, 14, 17]. Furthermore, as the landscape of cervical cancer biology continues to evolve, the discovery of new targets may facilitate the development of personalized medicine approaches, ultimately leading to more effective and tailored therapeutic strategies for patients suffering from this disease [9, 10, 12, 14, 17].

Our results strongly support GJB5 as a novel therapeutic target for cervical cancer based on compelling evidence from multiple analyses. Examination of TCGA data revealed a significant increase in *GJB5* mRNA expression in cervical cancer tissues compared to normal cervical epithelium, with high levels of *GJB5* correlating with poorer clinical outcomes, including reduced overall survival. Furthermore, single-cell RNA sequencing confirmed *GJB5* overexpression specifically within the malignant tumor cell population of cervical squamous cell carcinoma. GJB5 expression is also elevated in cervical cancer tissues from locally treated patients and in a panel of primary and established cervical cancer cells. Functional studies demonstrated that GJB5 shRNA or KO not only impaired the viability, proliferation, and migratory capacity of cervical cancer cells but also induced apoptotic processes. Conversely, forced overexpression of GJB5 led to enhanced malignant behaviors. Additionally, the knockdown of GJB5 resulted in a significant reduction in the growth of cervical cancer xenografts, reinforcing its potential as a promising target for therapeutic intervention in cervical cancer management.

Akt-mTOR signaling hyperactivation is a critical driver of cervical cancer malignancy and progression due to its pivotal role in regulating cell growth, proliferation, and survival [35, 38]. This pathway facilitates metabolic reprogramming, allowing cancer cells to sustain their energy demands and promote tumorigenesis [35, 38]. Moreover, hyperactivation of Akt-mTOR signaling has been implicated in enhancing resistance to apoptosis, thereby contributing to the aggressive nature of cervical cancer [35, 38]. The pathway also influences the tumor microenvironment by promoting angiogenesis and immune evasion, further facilitating cancer progression [35, 36, 43]. Despite the established importance of Akt-mTOR signaling in cervical cancer, the precise mechanisms underlying its hyperactivation in cervical cancer remain not fully understood.

In our investigations into the underlying mechanisms of cervical cancer progression, we showed that GJB5 is integral to the activation of the Akt-mTOR signaling pathway. Specifically, GJB5 knockdown or KO resulted in a marked reduction in the phosphorylation of both Akt and S6K1, indicating a significant impairment of the Akt-mTOR signaling cascade. Conversely, GJB5 overexpression was associated with enhanced Akt-mTOR signaling in primary human cervical cancer cells. Importantly, in GJB5-silenced cervical cancer cells, the restoration of Akt-mTOR activation through the expression of caAkt1 reversed the inhibitory effects on cell proliferation and migration. This finding underscores the critical link between GJB5 and Akt-mTOR signaling in cervical cancer malignancy.

Gαi proteins consist of three main subunits: Gαi1, Gαi2, and Gαi3. Gαi proteins are found at elevated levels in various human cancers and play a crucial role in tumor development and progression [30–33, 44]. Recent studies have emphasized the importance of Gαi1 and Gαi3 in facilitating signal transmission from multiple receptor tyrosine kinases (RTKs), including the epidermal growth factor receptor (EGFR), fibroblast growth factor receptor (FGFR), keratinocyte growth factor receptor (KGFR), stem cell factor receptor c-kit, brain-derived neurotrophic factor receptor TrkB, and vascular endothelial growth factor receptor 2 (VEGFR2) [30, 39, 41, 42, 45, 46]. These Gαi proteins are vital for mediating the oncogenic Akt-mTOR pathway by these surface receptors [30, 39, 41, 42, 45, 46]. In addition to their connections with RTKs, Gαi1 and Gαi3 are also known to interact with various non-RTK receptors. Notable examples include the leucine-rich repeat-containing G-protein coupled receptor 4 (LGR4) for R-spondin 3 (RSPO3), CD146 for Netrin-1, the interleukin-4 (IL-4) receptor, and the Toll-like receptor 4 (TLR4) for lipopolysaccharide (LPS) [40, 47–49]. Through these associations, Gαi proteins play a significant role in activating the Akt-mTOR signaling pathway, underscoring their importance as oncogenic drivers and their potential as targets for cancer therapies.

A recent study identifies Gαi3 as a potential oncotarget in cervical cancer, demonstrating that its elevated expression correlates with poor patient survival and enhanced cancer cell growth. Silencing or knocking out Gαi3 significantly inhibited these processes and reduced Akt-mTOR activation, suggesting its role in promoting cervical cancer progression [29]. This study identified a novel mechanism by which GJB5 promotes Akt signaling in cervical cancer. We demonstrated a physical interaction between GJB5 and Gαi3 through co-IP assays in both cervical cancer cells and fresh tumor tissues. Silencing Gαi3 significantly attenuated Akt phosphorylation in cervical cancer cells. Importantly, GJB5 overexpression failed to restore Akt activation in Gαi3-deficient cells, highlighting the critical role of Gαi3 in GJB5-mediated Akt signaling. Conversely, Gαi3 overexpression enhanced Akt phosphorylation in primary cervical cancer cells, and this effect was partially inhibited by GJB5 silencing. These findings reveal that GJB5 promotes Akt activation through a novel mechanism involving its interaction with Gαi3 in cervical cancer cells.

## Supplementary information

Original data set

Figure S1 and S2

## Data Availability

All data are available upon request.
