# Supplementary figures and images for: Gap junction protein beta 5 interacts with Gαi3 to promote Akt activation and cervical cancer cell growth
Source: Cell Death Dis. 2025 Jun 19;16(1):461. doi: 10.1038/s41419-025-07768-w (PMC12179280; doi:10.1038/s41419-025-07768-w)

Figure S1.

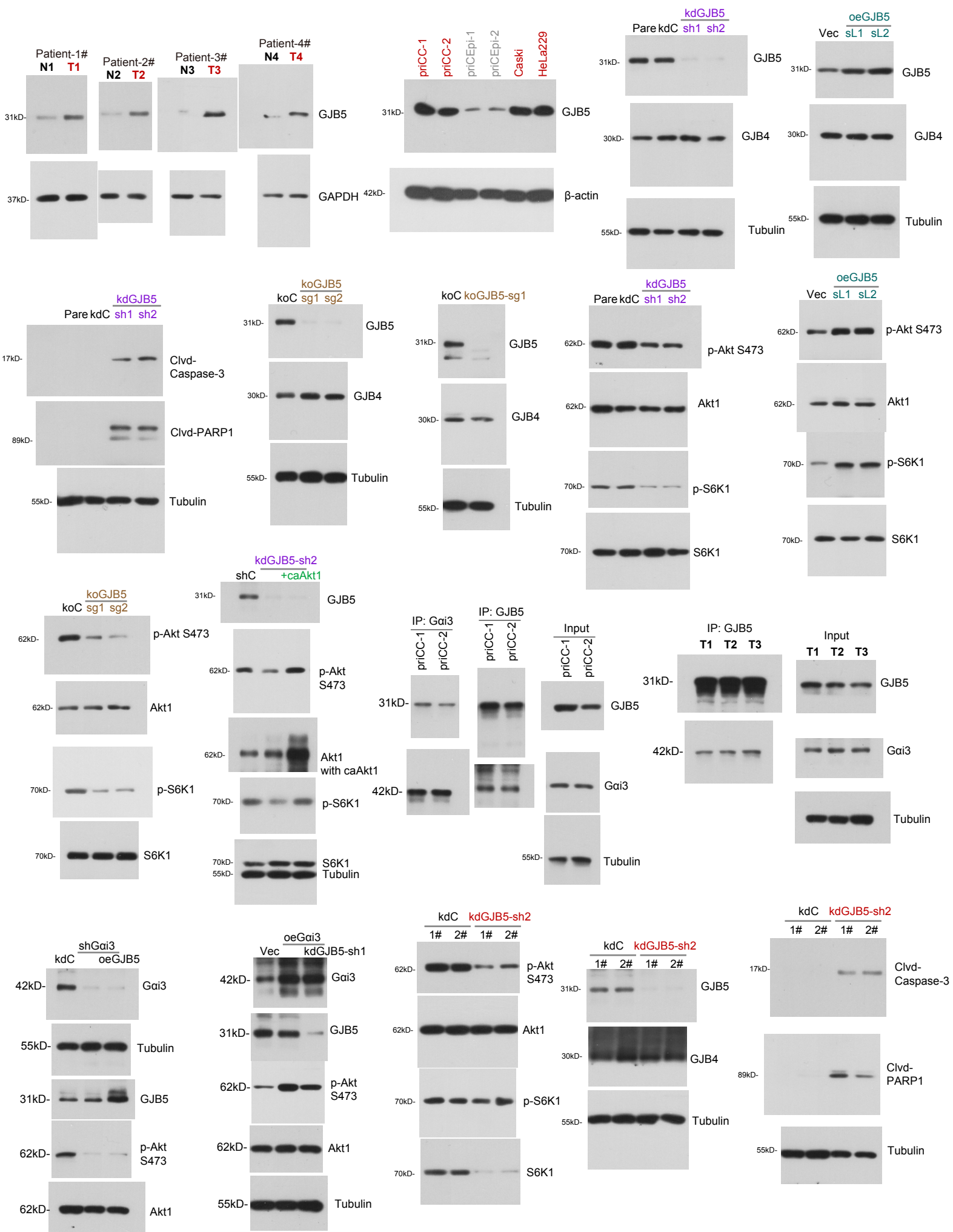

Figure S1: The un-cropped blotting images of the study.

Supplement: Supplementary file 1 — Original data set [file 41419_2025_7768_MOESM1_ESM.pdf]
