# Supplementary material for: Gap junction protein beta 5 interacts with Gαi3 to promote Akt activation and cervical cancer cell growth
Source: Cell Death Dis. 2025 Jun 19;16(1):461. doi: 10.1038/s41419-025-07768-w (PMC12179280; doi:10.1038/s41419-025-07768-w)
Supplement: Supplementary file 2 — Figure S1 and S2 [file 41419_2025_7768_MOESM2_ESM.pdf]

Figure S1.

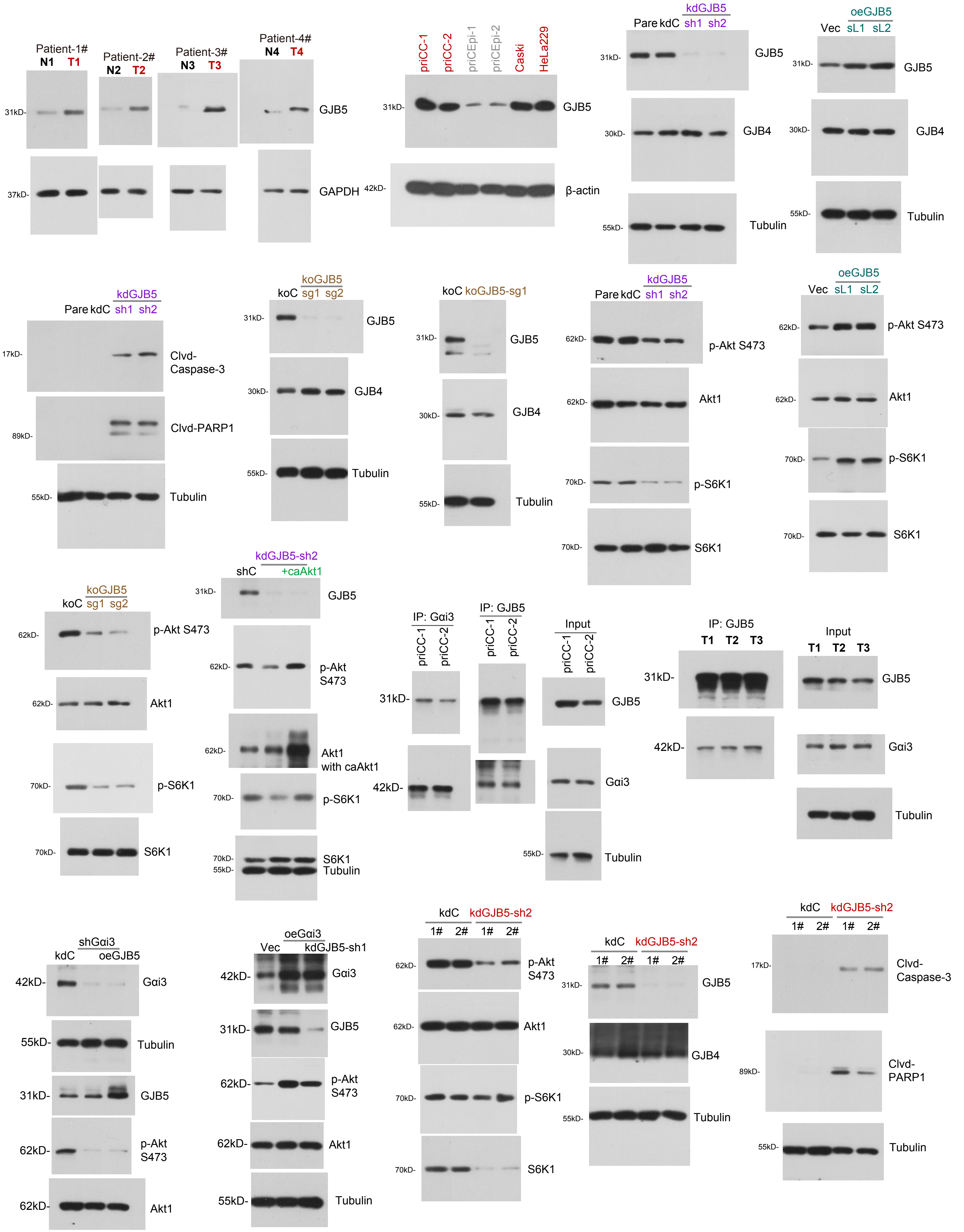

Figure S1: The un-cropped blotting images of the study.

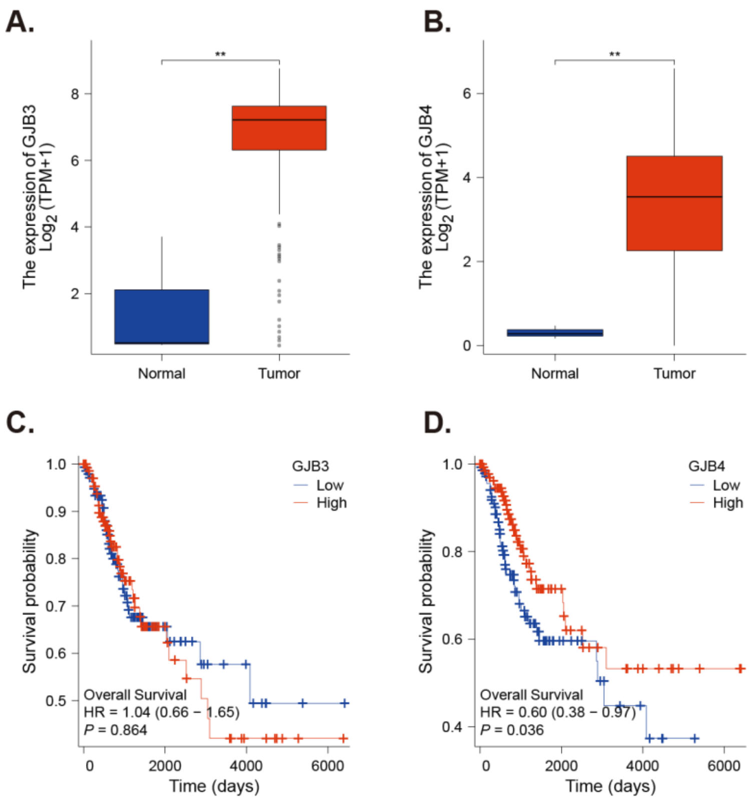

Figure S2. Box plots showing mRNA expression levels (Log2(TPM+1)) of *GJB3* (**A**) and *GJB4* (**B**)in normal tissues versus cervical cancer tissues. Kaplan-Meier curves for overall survival of cervical patients, stratified by high versus low expression levels of *GJB3* (**C**)and *GJB4* (**D**). Hazard Ratios (HR), 95% confidence intervals, and log-rank ***P***-values are shown. Asterisks (**) indicate ***P*** < 0.01.
